# Representation Wars: Enacting an Armistice Through Active Inference

**DOI:** 10.3389/fpsyg.2020.598733

**Published:** 2021-01-07

**Authors:** Axel Constant, Andy Clark, Karl J. Friston

**Affiliations:** ^1^Charles Perkins Centre, The University of Sydney, Sydney, NSW, Australia; ^2^Department of Philosophy, The University of Sussex, Brighton, United Kingdom; ^3^Department of Informatics, The University of Sussex, Brighton, United Kingdom; ^4^Department of Philosophy, Macquarie University, Sydney, NSW, Australia; ^5^Wellcome Trust Centre for Human Neuroimaging, University College London, London, United Kingdom

**Keywords:** philosophy of cognitive science, free energy principle, active inference, embodiment, representationalism

## Abstract

Over the last 30 years, representationalist and dynamicist positions in the philosophy of cognitive science have argued over whether neurocognitive processes should be viewed as representational or not. Major scientific and technological developments over the years have furnished both parties with ever more sophisticated conceptual weaponry. In recent years, an enactive generalization of predictive processing – known as active inference – has been proposed as a unifying theory of brain functions. Since then, active inference has fueled both representationalist and dynamicist campaigns. However, we believe that when diving into the formal details of active inference, one should be able to find a solution to the war; if not a peace treaty, surely an armistice of a sort. Based on an analysis of these formal details, this paper shows how both representationalist and dynamicist sensibilities can peacefully coexist within the new territory of active inference.

## Introduction

This paper proposes a way to end the representation wars. Focusing on recent formal developments, we aim to show that the concept of generative models as applied to the brain under active inference accommodates a representationalist and a dynamicist (a.k.a. non-representational) view of cognition. More precisely, we show that the architecture or configuration of neuronal pathways under a (Markovian) generative model (for discrete state spaces) can – and generally speaking will – realisze both representational and non-representational processes.

In Section 2 of this paper, to help readers unfamiliar with the notion of representation in the philosophy of cognitive science, we present a heuristic overview of its history, focusing on salient moments of its war over the past 30 years. Although this history is complex and more nuanced than what we can present here, we believe that this discussion evinces some of the motivations behind the notion of representation and its contestations. In Section 3, we present the architecture of generative models’ representationalist pathways. This allows us to segue into a discussion of dynamic pathways in Section 4. Section 5 discusses some worries. We then conclude in Section 6 with some brief remarks on good practice in the philosophy of cognitive science, when appealing to the mechanics of active inference.

Note that we do not engage with debates concerning active inference *per se*, nor do we venture into a philosophical justification of its use in cognitive neuroscience. Rather, we start from the premise that active inference is a suitable theory, as evidenced by the large literature that evidences, employs, argues for, and teaches its workings. For a comprehensive introduction and for a review of the formal fundaments and empirical evidence, we refer the reader to Beal (2003), Bogacz (2017), Buckley et al. (2017), Friston (2018), Keller and Mrsic-Flogel (2018), Parr et al. (2019).

Also, note that the argument presented in this paper is of a different kind than those currently available in the literature on representationalism in predictive processing and active inference (e.g., Clark, 2015a,c,b; Allen and Friston, 2016; Gładziejewski, 2016; Dolega, 2017; Kiefer and Hohwy, 2017), as it is not based on an intuitive conceptual analysis, but rather on a formal, analytic reading of the theory. The argument we present is simple. We show that if one agrees with the sufficient criteria for representationalism described in Section 2, then one is compelled to agree with the claim made in this paper; namely, that formally, representational and non-representational cognitive processes can be implemented by the brain under active inference. Given that active inference is a formal theory of functional neuroanatomy, the debates on the representational nature of brain processes concerning active inference should come to an end. Thus, the upshot of this paper is to move forward the philosophical debates on representationalism in active inference and to enable practical debates about the varieties of possible implementations of representational and non-representational neuronal processes.

## 30 Years of Representation Wars

### 1980s Connectionism

As the story goes, until the 1990s, driven by advances in computer science, the philosophy of cognitive science was dominated by cognitivism and connectionism (e.g., Fodor, 1975; Churchland, 1989). Connectionism was presented as a first attack on cognitivism – cognitivism being an attempt at understanding the brain as a logical symbol manipulating system. For connectionists, the brain should not be studied as a symbol manipulating system, but rather, consistent with the brain’s actual neurophysiology, as a set of hierarchically deployed neural networks. The spirit of connectionism is still very much alive today, such as in deep learning research (for a review see LeCun et al., 2015).

Both cognitivism and connectionism deal with a view of cognition as a problem-solving activity. And both paradigms have typically invoked some notion of representation, with the main difference being whether the representations had symbol-level content or something softer, something contentful yet “sub-symbolic” (for extensive discussion, see Clark, 1989, 1993).

Although these are different types of representations, each involving different criteria, a cognitive process will – for the purposes of this paper – be deemed representational whenever that process can be said to fulfill the following sufficient conditions (see Siegel, 2010; Hutto and Myin, 2013):

(i) The cognitive process is about something else (a.k.a. aboutness).
(ii) The cognitive process has satisfaction conditions with respect the thing it is about.

### 1990s Dynamicism

The 1990s marked the rise of embodied views in cognitive science such as enactivism (Varela et al., 1991) and radical embodied cognition (Chemero, 2009). Embodied approaches were motivated by developments in the field of dynamical system theory, which casts cognitive systems as coupled quantitative variables, mutually changing interdependently over time (Van Gelder, 1995; Thelen and Smith, 1996; Beer, 2000); one variable being the organism, the other being the environment. Dynamicism has been driven by two main criticisms of much previous work (Thompson, 2007):

(1) Since the brain is embodied, we cannot abstract cognition from the body, and consequently from the environment;
(2) Since representationalism posits the mediation of the world and cognition by the mental manipulation of representations, representationalism cannot genuinely acknowledge embodiment.

Therefore, for these kinds of dynamicists – see Clark (1997) for a more liberal approach – we should reject the representational view of cognition altogether. Instead, cognition should be viewed as a process of self-organization among the components of the biological system performing the cognitive activity. These components include the brain (internal states) and the body and the environment (external states). On that view, cognition is a homeostatic and allostatic process of attunement to cope with environmental perturbations; a process of “coping, not computing.”

### 2000s Active Inference Westphalia?

At the turn of the millennium, based on a Helmholtzian view of embodied perception, the theory of active inference was introduced as a realization of the free energy principle (Friston et al., 2006, 2016; Friston, 2010). This enactive generalization of predictive processing marked a paradigm shift in cognitive science: active inference became a potential candidate to meet the challenge of the grand unification of neurocognitive functions (Clark, 2013). Since then, many enthusiasts have leveraged active inference to attempt explanations of the underlying computational processes of biobehavioral functions such as action, perception, learning, attention, memory, decision making, emotions, planning and navigation, visual foraging, communication, social learning, and many more (Feldman and Friston, 2010; Joffily and Coricelli, 2013; Friston and Frith, 2015; Friston et al., 2016; Mirza et al., 2016; Parr and Friston, 2017b; Constant et al., 2018a,b; Kaplan and Friston, 2018; Badcock et al., 2019).

In line with much of Bayesian statistics, active inference claims that the brain is fundamentally in the business of finessing a generative model of the causes of its sensations; as if the brain was a scientist, trying to infer the causal architecture of its own relation to its world. Put another way, under active inference, the brain is a dynamical system that models the action-relevant causal structure of its coupling with the other dynamical system that embeds it – the body and the environment (i.e., the system generating its sensations). The mathematical formalism of active inference describes neuronal dynamics as a gradient flow that optimizes the evidence for a generative model of the lived world. On this view, neuronal networks embodied by the brain form a set of nodes (modeling hidden states) and edges (modeling conditional dependencies) of a probabilistic (Bayesian) graphical model.

But active inference itself soon became contested ground too. The cognitivist campaign claimed that “brains as generative models” were “rich and reconstructive, detached, truth-seeking inner representations” of the world (Hohwy, 2013, 2016); others [such as Clark (2015c)] resisted by claiming that generative models were in fact manifest as transient webs of neuronal coupling that are cost-efficient, and sometimes (though not always) freed from heavy-duty manipulation of internal representations – generating actions that exploited environmental opportunities by weaving themselves closely to the opportunities provided by body and the world (Clark, 2013, 2015c).

## Representational Pathways in Active Inference

Active inference assumes that the brain entails^1^ a causal model of the world (a.k.a. a generative model), whose structure represents the components involved in the cognitive function of interest, as well as the dynamics that realize that cognitive function. Formally, these components and dynamics are expressed as a Bayesian graphical model, with nodes and edges representing the dynamic relations among components, and the structure of which is assumed to map onto the neuroanatomy of neuronal systems realizing any cognitive function. The cognitive function, then, is realized by these dynamics – that play the role of neuronal message passing in the service of belief updating (i.e., inference) that underwrites the cognitive function in question (Friston et al., 2017a).

The representational interpretation of active inference is employed to study cognitive functions that rely on dynamics and components of the generative models that involve the internal manipulation of representational content (e.g., beliefs about hidden states of the world,^2^ including one’s body and physiology) (Hohwy, 2013, 2019). The motivation for appealing to representational generative models to explain perception and action in active inference stems from the inverse nature of the dual inference problems our brains solve (i.e., figuring “what causes what” before inferring “what caused that”):

(i) Perceptual problem: The brain does not have direct access to causes of sensations, nor is there a stable one-to-one mapping between causes and sensations. For instance, a sensory input (e.g., red sensation) may be caused by multiple fluctuating causes (e.g., red jacket, red car, red traffic light). In the philosophical literature, this problem is sometimes referred to as the black box, seclusion or solipsism problem (Clark, 2013; Hohwy, 2016; Metzinger and Wiese, 2017).
(ii) Action planning problem: All the brain can work with are the sensory inputs it receives. If we are to engage adaptive action, we must not only infer the causes of our sensations (i.e., forming a sufficiently veridical perception – or conception – of the world in which we currently find ourselves), but we must also predict the consequences of engaging in this or that action in the future. In the philosophical literature, this problem is sometimes referred to as the problem of mere versus adaptive active inference (Bruineberg et al., 2016; Kirchhoff et al., 2018), and requires action planning (c.f., planning as inference in machine learning).

This means that under active inference, agents like us must find a solution to infer, in an ill-posed setting, both the nature of the cause of our sensations (e.g., the jacket, the traffic light, or the car), and to infer what action will lead to outcomes that are consistent with our model of the lived world (e.g., being on the other side of the street vs. under the wheels of a car). Under active inference, perception and action are explained as solutions to these inverse problems – crucially, solutions that rest upon optimizing exactly the same quantity, as we will see below.

### Perception

Formally, the problem of indirect perception can be approached as follows. Consider a sensory outcome _*o*_ generated by a hidden state (_*s*_). Taken together, these can be viewed as forming a joint probability distribution (_*P(s,o)*_). The only quantity to which the brain has access is a sensory consequence, not its cause. To perceive things, the brain must reconstruct the hidden state or cause (_*s*_), or rather its posterior probability; i.e., the probability of the cause, after observing the sensory datum _*P(s—o)*_.

Definitions of the constructs in this paper can be found in Table 1 of Friston et al. (2016), Table 2 of Da Costa et al. (2020), and in the Supplementary Information of Hesp et al. (2019). A conceptual description of the technical notions employed in this paper can be found in Box 2 of Veissière et al. (2020) – reproduced here for convenience. We refer the reader to these resources because the model considered in this paper rests on a standardized formalism that has been detailed elsewhere. Note that the model we present here can be understood solely on the basis of a narrative description, and thus, can be viewed as playing an iconic role.

**FIGURE 1:**
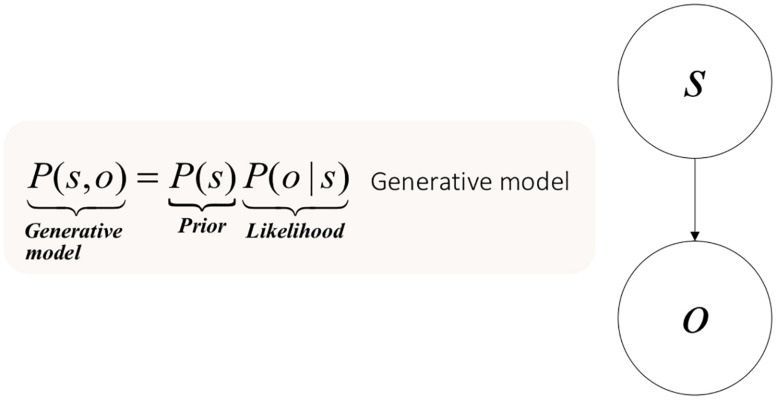
Elementary generative model for perception and the problem of indirect inference.

To infer this posterior probability, the brain learns the causal (i.e., generative) model of the manner in which the world caused the sensation. Learning here is a technical term. It refers to the optimization of the parameters of a model – here the generative model. The brain learns the parameters of hidden states causing sensory outcomes; about which the brain may have prior beliefs. These prior beliefs are part of the generative model entailed by the brain. Hence, a generative model decomposes into prior beliefs about hidden states and a likelihood of these hidden states, given outcomes. One can easily follow this decomposition by visualizing the graphical model that makes up the generative model in Figure 1.

In an ideal scenario, the brain could use Bayes rule to infer the true probability of the cause, by using the probability of the data, known as model evidence or marginal likelihood:

(1) $$P\,\left(s|o\right)=\frac{P\,\left(s\right)\,P\,\left(o|s\right)}{P\,\left(o\right)}$$

The marginal likelihood _*P(o)*_ refers to the probability of sensory data averaged – or marginalized – over all possible hidden states:

(2) $$P\,\left(o\right)=\sum_{s}P\,\left(s,o\right)$$

To represent the marginal likelihood and perform exact inference (as in Equation 1), the marginalization that the brain would have to perform would be intractable, as there may be a near infinite number of causes with various probabilities for each sensory datum. This is at the core of the inverse problem of inference; direct calculation of the posterior probability of one’s beliefs given sensory data _*P(s—o)*_ is simply intractable. Thus, the problem of indirect inference may be restated as follows: the brain cannot access the true posterior probability over the causes of its sensations because this requires evaluating an intractable marginal likelihood. What the brain can do, however, is to perform “approximate Bayesian inference” based on its prior beliefs and the sensory data it receives.^3^ In active inference, the “manipulation of content” rests on this method of inference known as approximate Bayesian inference (Feynman, 1972; Dayan et al., 1995; Beal, 2003).

Approximate Bayesian inference allows the inversion of the generative model to estimate the marginal likelihood via an approximation to the true posterior over sensory causes (i.e., what the brain would do using exact Bayesian inference if it had access to the marginal likelihood). Taking advantage of Jensen’s inequality, the method of approximate Bayesian inference involves the minimization of an upper bound on (negative log) model evidence (a.k.a. surprisal), called variational free energy. This bound is constructed by using an arbitrary probability distribution^4^_*Q(s)*_ that is used to minimize the variational bound – and the generative model _*P(s,o)*_ :

(3) $$\begin{array}{ccc} F & = & \sum Q\,\left(s\right)\,\ln\frac{Q\,\left(s\right)}{P\,\left(s,o\right)}\\ & = & \sum Q\,\left(s\right)\,\ln\frac{Q\,\left(s\right)}{P\,\left(s|o\right)}-\ln P\,\left(o\right)\\ & = & E_{Q\,\left(s\right)}\,\left[\ln\frac{Q\,\left(s\right)}{P\,\left(s|o\right)}\right]-\ln P\,\left(o\right)\;\\ & = & D\,\overset{\text{Bound}}{\overbrace{\left[\underset{\begin{array}{c} \text{Approximate}\\ \text{posterior} \end{array}}{\underbrace{Q\,\left(s\right)}}||\underset{\begin{array}{c} \text{True}\\ \text{posterior} \end{array}}{\underbrace{P\,\left(s|o\right)}}\right]}}-\ln\underset{\begin{array}{c} \text{Marginal}\\ \text{likelihood} \end{array}}{\underbrace{P\,\left(o\right)}} \end{array}$$

Equation 3 says that the free energy of our approximate posterior (i.e., Bayesian) beliefs, given some sensory outcomes, is the Kullback–Leibler divergence (_*D*_) from the true posterior probability of external states, given the sensory input; minus the (negative log) marginal likelihood. Estimating the marginal likelihood can be achieved by minimizing the free energy functional of (Bayesian) beliefs and sensations:

(4) $$\begin{array}{ccc} Q\,\left(s\right) & = & \underset{s}{\arg\min}F\Rightarrow \\ & & Q\,\left(s\right)\approx \underset{\mathrm{True}\,\mathrm{posterior}}{\underbrace{P\,\left(s|o\right)}}\\ & & F\approx -\underset{\mathrm{Log}\,\mathrm{evidence}}{\underbrace{\ln P\,\left(o\right)}} \end{array}$$

The Kullback–Leibler (KL, or _*D*_ here) divergence represents the difference between the agent’s beliefs about external states _*Q(s)*_, and the true posterior probability over these states, given the sensory data _*P(s—o)*_. Any KL-divergence is always non-negative, which means that as the free energy gets smaller (i.e., as we minimize the functional) the divergence tends toward zero. This means that minimizing free energy entails:

Marginal Likelihood Estimation (a.k.a. MLE, Beal, 2003) by making free energy a tight upper bound on the (negative log) marginal likelihood _–ln P(o)_.

Perception (and learning) of external states by making the approximate posterior _*Q(s)*_ a good approximation of the true posterior _*P(s—o)*_.

Perception (and learning), then, is simply the process whereby the approximate posterior _*Q(s)*_ – parameterized or encoded by the internal states of the brain – are made “statistically consistent” with the true posterior distribution over the external states of the world given sensory observations.

Note that there is some debate as to whether the reduction of the Kullback–Leibler divergence is a representational process (Kirchhoff and Robertson, 2018). Whether this process is representational or not, the probability distributions it manipulates are most certainly instances of representations (cf. Badcock et al., 2019). The divergence between two probability distributions can be said to be “right” or “wrong” with respect to some satisfaction conditions (i.e., a reducing divergence is better than an increasing divergence). Therefore, even if the process *per se* (i.e., reduction of the divergence or evidence bound) is non-representational, the components involved in this process make that process one of “manipulation” of representations. A similar theme is seen in Bayesian decision theory, game theory and economics where the evidence bound can be interpreted as leading to bounded rationality (i.e., approximate Bayesian inference) (Friston et al., 2013). The rationality of decisions again speaks to an inherent representationalism that underwrites the “right” sort of decisions.

Now, depending on the structure (i.e., entailed knowledge) in the generative model, approximate Bayesian inference not only optimizes beliefs about the world “out there” but also beliefs about the consequences of doing this or that. These beliefs yield inference to the best action to engage (see below). As we have seen, in the case of perception, approximate Bayesian inference involves minimizing free energy, which is an upper bound on (negative log) marginal likelihood. We now turn to action planning as another instance of representational cognitive process.

### Action Planning

**FIGURE 2:**
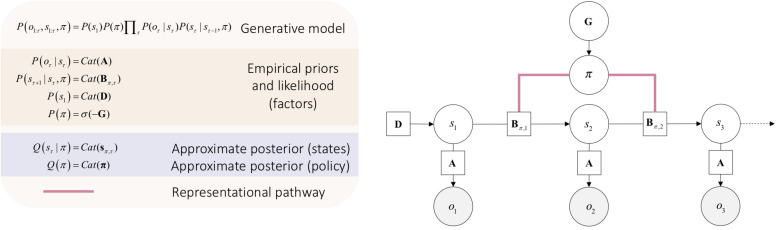
Minimal discrete state space generative model for action. Open circles are random variables (hidden states and policies). Gray filled circles are observable outcomes. Squares are known variables, such as the model parameters. _Cat_ refers to categorical distributions. The equations in the beige box (upper left) specify the architecture of the generative model (for a complete description see Friston et al., 2017b). The likelihood matrix _*A*_ specifies the probability of outcomes for each combination hidden states _*s*_. The novelty of this generative model rests on the addition of a policy _π_ that determines state transitions, represented by the policy-dependent transition matrix *B*_π,τ_. The initial state is specified by _*D*_. The approximate posterior of the future hidden state *s*_π,τ_ at time _*t*_ relative to the policy _π_ is found by evaluating the approximate posterior for the policy _*Q(π)*_. This policy will be the one with the least expected free energy _*G=–lnP(π)*_ that determines prior beliefs about the policy being pursued; namely _*P(π)*_ that can recovered from expected free energy, using the softmax operator _σ_ (for a complete description see Friston et al., 2017a). Note that the edges that link the policy and the transition matrices are undirected. This is important, as it means that the evaluation of expected free energy requires a message from hidden states representations, thereby affording a representationalist pathway.

To account for action, one must start thinking about the manner in which states of the world change over time. This requires us to cast the generative model over multiple times steps τ, into the future and how an action policy π (i.e., possible sequence of actions) may influence these the trajectory of states when this or that policy is realized. Thus, our generative models will have the form _*P(s,π,o)*_ – to allow us to infer future hidden states and associated outcomes *o*_τ_ relative to a policy π (see Figure 2). Here, for the sake of simplicity, we will focus on a discrete formulation of the ensuing generative model for action.

The structure of the graphical model in Figure 2 allows us to work with a free-energy appropriate for outcomes that have yet to be observed. This is known as expected free energy G (see Parr and Friston, 2017a):

$$G\,\left(\pi ,\tau \right)=\sum P\,\left(o_{\tau }|s_{\tau }\right)\,Q\,\left(s_{\tau }|\pi \right)\,\ln\frac{Q\,\left(s_{\tau }|\pi \right)}{P\,\left(o_{\tau },s_{\tau }|\pi \right)}$$

$$=\sum P\,\left(o_{\tau }|s_{\tau }\right)\,Q\,\left(s_{\tau }|\pi \right)\,\ln\frac{Q\,\left(s_{\tau }|\pi \right)}{P\left(s_{\tau }|o_{\tau },|\pi \right)P\left(o_{\tau }\right)}$$

$$=\sum P\,\left(o_{\tau }|s_{\tau }\right)\,Q\,\left(s_{\tau }|\pi \right)\,\ln\frac{Q\,\left(s_{\tau }|\pi \right)}{P\left(s_{\tau }|o_{\tau },|\pi \right)}$$

$$-\sum P\,\left(o_{\tau }|s_{\tau }\right)\,Q\,\left(s_{\tau }|\pi \right)\,\ln P\,\left(o_{\tau }\right)$$

$$=E_{P\,\left(o_{\tau }|s_{\tau }\right)\,Q\,\left(s_{\tau }|\pi \right)}\,\left[\ln\frac{Q\,\left(s_{\tau }|\pi \right)}{P\,\left(s_{\tau }|o_{\tau },\pi \right)}\right]$$

$$-E_{P\,\left(o_{\tau |s_{\tau }}\right)\,Q\,\left(s_{\tau }|\pi \right)}\,\ln P\,\left(o_{\tau }\right)$$

$$=\underset{\text{Instrumental}}{\underbrace{-E_{Q\,\left(s_{\tau |\pi }\right)\,P\,\left(o_{\tau }|s_{\tau }\right)}\,\left[\ln P\,\left(o_{\tau }\right)\right]}}$$

(5) $$+\underset{\text{Epistemic}}{\underbrace{E_{Q\,\left(s_{\tau |\pi }\right)\,P\,\left(o_{\tau }|s_{\tau }\right)}\,\left[\ln Q\,\left(s_{\tau |\pi }\right)-\ln P\,\left(s_{\tau |o_{\tau },\pi }\right)\right]}}$$

In Equation 5, expected free energy of a policy at a given time _*G(π,τ)*_ decomposes into a pragmatic or instrumental term and an epistemic term, also known as extrinsic and intrinsic values. The pragmatic term, or extrinsic value constitutes the goal seeking component of expected free energy (often referred to as expected value or utility in psychology and economics) (Kauder, 1953; Sutton and Barto, 1998). Extrinsic value is the expected value of a policy relative to preferred outcomes that will be encountered in the future _lnP(o_τ)_. In turn, the epistemic term, or intrinsic value constitutes the information seeking component of expected free energy. Intrinsic value is the expected information gain relative to future states under a given policy (i.e., “what policy will best guarantee the minimization of uncertainty in my beliefs about the causal structure of the world?”). In visual neurosciences, this is called salience and is a key determinant of epistemic foraging or exploratory behavior (Itti and Baldi, 2009; Sun et al., 2011). As such, it is sometimes referred to as intrinsic motivation (Ryan and Deci, 1985; Oudeyer and Kaplan, 2007; Schmidhuber, 2010; Barto et al., 2013); Schmidhuber, 2010). Selecting the policy that affords the least expected free energy guarantees an adaptive action, that is, that first consolidates knowledge about the world, then optimizes – i.e., works toward – preferred outcomes. For a complete discussion see Friston et al. (2015).

### Summary: The Reason Why Perception and Action Planning Rest on Representational Processes

In summary, under active inference, action selection is a process of manipulating representations about future states of the world to maximize one’s knowledge and secure desired (predicted) outcomes and sensory encounters. This inference or belief updating about “what I am doing” rests on perceptual inference. Perception, in turn, is a process of updating mental representations of states of the world and their relationship to sensory consequences, so as to make these representations as consistent as possible with the true state of the world. Hence, more generally, perception and action planning, under active inference, are instances of representational processes. The statistical structure of the likelihood mapping tells me that the most likely cause of the sensory entry is the cause that my belief represents; and put bluntly, minimizing uncertainty in beliefs is for the most part what “forming a percept” is about. In turn, action selection is an inference process that relies on these optimized beliefs about sensory causes, and the consequences of future moves in a rich and reconstructive fashion. Action selection tells me that since I am a surprise or free energy minimizing creature, I should selectively engage with the world to minimize expected surprise or uncertainty. This requires me to respond to epistemic affordances – to resolve uncertainty – while securing familiar (i.e., *a priori* preferred) sensory outcomes. This will minimize my uncertainty about future states and maximize the utility of my action.

In active inference, the need for rich, representations involving generative models stems directly from the problem of inverse inference about causes and adaptive actions to resolve uncertainty about those causes. The ill-posed nature of the inference problem we face forces us to first “figure out for ourselves” “what causes what?” before being able to zero-in on “what caused that” (perception), and “I will cause that” (i.e., action planning). This problem forces us to learn hierarchically (i.e., over multiple levels of prior beliefs) and temporally (i.e., over multiple time steps, such as in Figure 2) deep generative models (Friston et al., 2017c).

## Dynamic Pathways in Active Inference

We turn now to the role of non-representational dynamics in active inference. There is a technical sense in which an austere, dynamicist reading of active inference is licensed in a fundamental way. This follows because the representational account above emerges from a certain kind of dynamics; namely, gradient flows on variational and expected free energy (cf. Ramstead et al., 2019). In other words, the cognitivist functionality rests upon optimizing free energy and this optimization is a necessary consequence of neuronal dynamics that – not unlike a river flowing downhill – descend free energy gradients – to find free energy minima where the gradients are destroyed (Tschacher and Haken, 2007). Indeed, the back story to active inference shows that this kind of dynamical behavior is a necessary aspect of any self-organization to nonequilibrium steady-state in any random dynamical system that possesses a Markov blanket (Friston, 2013). On this view, any system that possesses some attracting states has dynamics that look “as if” they are trying to minimize free energy and therefore acquire a representational and teleological interpretation (cf. Ramstead et al., 2019).

While there are interesting issues that attend the distinction between a purely dynamical formulation of active inference – and a representationalist reading in terms of dynamics and information geometry – we will consider non-representationalist formulations. These formulations speak to notions of extended and embedded optimization, which call upon hierarchical dynamics that consider the dynamical exchange between an agent and its (physiological, evolutionary, and cultural) econiche. Accordingly, the dynamic pathways in generative models under active inference – examples appear below – do not appeal to the manipulation of representations of hidden states of the world to explain the cognitive processes underlying the behavior they generate. Dynamic pathways can be exemplified by application to a specific class of unplanned action – more specifically enculturated action (more on that below) – that does not rest on the manipulation of rich webs of internal representations. Rather, heuristically, the dynamicist view is a view of action that only requires processing “something as doing,” such that the “doing” (e.g., action sequences or realized policies) is directly conditioned upon the “something” (e.g., sensory observation). In the recent literature on active inference, this sort of action has been coined “deontic” action (Constant et al., 2019).

### Deontic Action

Deontic actions are actions for which the underlying policy has acquired a deontic value; namely, the shared, or socially admitted value of a policy (Constant et al., 2019). A deontic action is guided by the consideration of “what would a typical other do in my situation.” For instance, stopping at the red traffic light at 4 am when no one is present may be viewed as such a deontically afforded action.^5^

**FIGURE 3:**
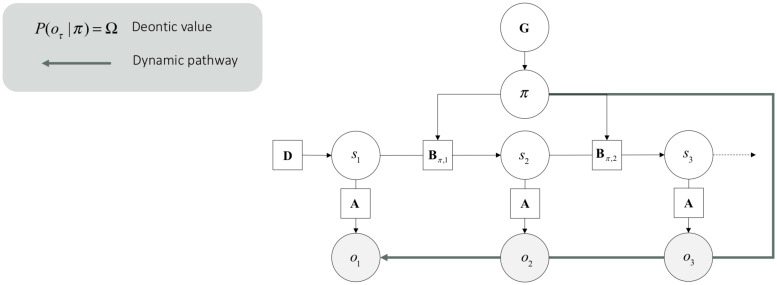
Dynamic pathway. This graphical generative model is the same as the one presented in Figure 2. However, it incorporates deontic value, which is read here as a dynamic pathway.

Central to our agenda, deontic actions are processed through different mappings in the generative model. Technically, deontic value is the likelihood of a policy given an observation _lnP(o_τ —π)_ that grounds posterior beliefs about policies.^6^ This likelihood is an empirical prior which constitutes expected free energy. The deontic value _lnP(o_τ —π)_ effectively supplements or supplants the likelihood of outcomes under different states _*P(o_τ —s_τ)*_ (see Figure 3). From the point of view of the generative model, this means that if I am pursuing this policy then these outcomes are more likely (e.g., when I stop doing something, I am likely to see a stop sign). From the point of view of inference, this means that if I see these deontic outcomes, I will infer I am doing this (e.g., if I see a stop sign, I will stop). Put simply, a deontic action is an available (i.e., plausible) policy that is triggered by a sensory input, and which leads directly to an internally consistent action. Crucially, this means that deontic action selection bypasses representational beliefs about states of the world and associated sensory consequences.

The computational architecture of deontic action is a clear candidate to implement a form of dynamicism under active inference. In effect, the information processing underlying deontic action eludes the two sufficient conditions of representationalism presented in Section 2:

(i) Since they involve the inversion of a policy-outcome mapping, instead of state-outcomes mappings, deontic processes do not entail a propositional attitude involving the mediation of manipulations of one’s (Bayesian) beliefs standing for sensory causes in the world. Deontic processes do not have “aboutness.”
(ii) Success conditions in epistemology are about the way an agent’s act (e.g., an assertion, or another kind of speech act) needs to relate to the states of the world toward which it is directed for it to work. This means that usually, what the agent (or her brain) is seeking to optimize isn’t the issue. Under active inference, however, one ought to consider active brain processes and success conditions that do not relate to the external world *per se.* Brain processes can arbitrate between successful or unsuccessful alternatives with respect to the internal generative model *per se*, such as in the case of action planning, where action policies are compared in terms of the free energy – under the generative model – expected in the future. This means that success conditions can be given with respect to the generative model *per se*, not the world generating the observation (a.k.a. generative process). This is a subtle, yet crucial point, which becomes apparent when considering the probability distributions involved in various inference processes in the brain. For instance, when inferring hidden states (i.e., perceiving), the true posterior (*P*) approximated by the approximate posterior (*Q*) is the true posterior probability of the agent’s beliefs, not of the cause of the agent’s observations. “Getting it right,” in that case, again, is about getting it right with respect to one’s own beliefs; e.g., successfully exploring the state space of one’s own model of the world. This means that under active inference, there are two layers of success involved, one defined over the model, and one defined over the agent-world coupling (which corresponds to a more traditional epistemological point of view).

The second layer allows us to know when a generative model isn’t fit for purpose – e.g., in cases of mental disorders, where behavioral outcomes are maladaptive with respect to the individual’s environment. Those two layers of success are apparent in the fact that one can perform (Bayes) optimal inference while generating suboptimal behavior because of suboptimal (prior) beliefs (Corlett and Fletcher, 2014). Deontic processes conform to success conditions of this second layer. They do not have success conditions *qua* brain processes, but rather have success condition *qua* agent-world coupling processes. They are simple observation-action loops; not rich and reconstructive policy selection loops. This is so because deontic processes circumnavigate the computation of expected free energy, which as we have seen, is used to compare different policies with respect to their ability to maximize instrumental and epistemic values. Under active inference, the maximization of these values such as captured by expected free energy (*G*) is the success condition of non-deontic action selection in the brain. This construction means that inferences about states of the world – that admit a representationalist interpretation – are now replaced by direct action, without any intervening inference or representation of the consequences of action.

Having said this, the dual aspect architecture in Figure 3 means that the representationalist and dynamic pathways can happily live side-by-side, mutually informing each other – but both are sufficient for enactive engagement with the niche on their own. The distinction between the pathways – or routes to (subpersonal) action selection have some important implications. For example, deontic action circumnavigates expected free energy and therefore precludes planning as (active) inference. This means that under active inference, systems employing deontic strategies do not need to plan courses of action into the future. They simply act on the basis of the observation. Furthermore, in the absence of inference about hidden states, there could be no phenomenal opacity.^7^ For instance, this speaks directly to the sort of actions experts perform (e.g., athletes), which are often complex, though, for which experts do not seem to plan ahead (i.e., “in the head”). Such skilled actions seem to yield very little phenomenal opacity (e.g., as when the athlete responds, “I don’t know, I just did it in the flow of action,” or “we simply executed the game plan,” when interviewed about her game winning shot).

### Deontic Action as a Reflex?

The sort of “automatized” deontic behavior underwritten by dynamic pathways in the generative model might strike one as being conceptually close to the sort of cognitive processes underlying reflexes and other (homeostatic) functions processed through the autonomic nervous system. The computational pathway of deontic action indeed looks very much like a close control loop – secured by robust causal regularities in the world generating reliable sensory inputs – akin to a reflex processed at the brainstem and spinal cord level, but this time, processed in a “constructed local world” (Constant et al., 2019; cf. Ramstead et al., 2016). Under active inference, motoric and autonomic reflexes are framed as an action that manages the sensory signal that comes from within the system that generated it; e.g., suppression of interoceptive prediction error (Pezzulo et al., 2015).

Autonomic reflexes facilitate homeostatic regulation by engendering series of events necessary for the activity of the agent; e.g., salivation facilitates ingestion by easing the passing of the food. In this sense, they can be regarded as allostatic in nature. Similarly, one can think of sequences of deontic actions that facilitate social, affective, and emotional regulation; e.g., the outcome generated by the red traffic light triggers a stop, which facilitates reaching in my pocket to grab my phone to check my notifications (which itself might trigger salivation). For some enculturated agents, such a sequence of “social reflexes” may be necessary to pass through the day.

Now, the reader might worry that deontic action ends up being as unexciting as “digestive cognition.” But rest reassured, deontic action has been used to account for complex behavioral phenomena like social conformity: a.k.a., deference to the socially approved norm learnt through social influence or learning (Asch, 1955), and cooperative decision-making: a.k.a. decision-making under fairness psychology – as evidenced by the human tendency to zero in on fair decisions in economic games when compared to non-human animals (for a review see Henrich, 2015). Deontic action – as a social reflex – facilitates social interactions by easing the coordination among humans, if you will.

Deontic action is explained in terms of the circular causality between outsourcing decision-making to trusted others in the form of deontic cues (material or agential) – indicating the locally adaptive action – and learning the underlying cue-policy mappings. The “closed” control loop, then, comprises the enculturated agent and regularities in her (social) environment. In effect, deontic cues are defined as such because they represent a reliable informational aggregate of “what would a creature like me would do in this situation.” These cues consolidate over development and through the modification of the environment by generations of other enculturated agents (i.e., creatures like me) (Constant et al., 2019).

Once the action afforded by these cues is learnt, there is no need for computing future states and associated outcomes; these are secured by the configuration of the cultural setting. For instance, in Canada, you can trust stopping or crossing, according to the deontic cue afforded by the traffic light – because the traffic light has come to represent what others typically do at an intersection – perhaps not in France though. And when faced with an uncertain outcome in an economic game (e.g., “if I don’t know what the opponent will do and my reward depends on her response, should I share or should I maximize my gain?”), you can trust that the fair option is the one the other is most likely to select since you’ve been socialized as a “typical other,” presumably, just as the other did (for a review see Veissière et al., 2019).

Note that there is nothing new to the idea of reflex-like complex behavior. There is a long history of well-known concepts in cognitive psychology that covers what is at stake in the notion of deontic action (e.g., fast vs. slow thinking, autonomic vs. controlled processing, or the reflex arc of pragmatist psychologists). While we do not have the space to elaborate, one could note that contribution of deontic action to cognitive psychology represents only a small formal reinterpretation of the active inference framework. Further work should be done to anchor the notion of deontic action into its rich intellectual heritage.

### Summary: Deontic Actions Rest on Dynamic Processes

In summary, for proponents of dynamicism, generative models are not rich and reconstructive internal models. Rather, they are fast and frugal. If internal representations play a role at all, that role is thin and simple. As we have seen above, a rich and reconstructive internal model is one in which multiple trajectories of hidden states (with different precisions – more on that below) would be entertained before selecting the action. The fast and frugal alternative is the one that underwrites deontic action. Hence, for enculturated, deontically constrained agents like us, “what may often be doing the work [in generative models] is a kind of perceptually maintained motor-informational grip on the world: a low-cost perception-action routine that retrieves the right information just-in-time for use, and that is not in the business of building up a rich inner simulacrum” (Clark, 2015c, p. 11). This low-cost perception action routine corresponds to the web of deontic, or dynamic pathways learnt through enculturation (Constant et al., 2019).

## Worries About Rich Settings for Shallow Strategies?

Although computationally viable under active inference, our description of fast and frugal dynamic pathways based on deontic value might still raise some conceptual worries. In this section, we provide a brief discussion of some such worries.

### First Worry

One might worry that even deontic actions have to be selected through inferring the current context. The agent might need first to figure out if the context renders deontic action the most apt response. This worry raised by representationalists [e.g., Hohwy (2019)] might be a problem for the kind of account developed in this paper, and elsewhere [for example, Clark (2015a)]. For even the selection of frugal dynamic strategies would require the on-going inference afforded by a rich inner model, able to determine when such strategies are warranted – and override them when necessary. In other words, the recruitment of the right transient webs of deontic activity, at the right time, is itself a high-grade cognitive achievement where the inner model plays, representationalists argue, a necessary and ongoing role. The upshot is a worry that truly ecumenical accounts may be hostage to “a potential tension…. between allowing and withholding a role for rich models” (Hohwy, 2019). For surely (so the argument goes) the active inference agent must repeatedly infer when she is in a situation where some low-cost deontic response is viable. In effect, according to representationalists, setting and learning the confidence of prior beliefs through perceptual processes – such as described in Section 2 (a.k.a. precision, or gain control on sensory evidence, or prediction error) – needs to be a principled response, and that implicates the rich inner model even when the selected strategy is itself a frugal one.

In active inference, the mapping between causes (e.g., states and policies) and consequences (e.g., sensory outcomes) are parameterized in terms of probabilistic mappings that necessarily have a *precision*. In other words, the contingencies implicit in likelihood mappings can have different degrees of reliability, ambiguity, or uncertainty. For instance, if my child starts running toward the sea, as she gets further away (and closer to the water), my beliefs about whether she is in danger of drowning will become increasingly imprecise. Then, to disambiguate (hidden) states of affairs, I might plan an epistemic, representational strategy: running after my child to ensure she doesn’t go into the water without supervision. Had I known that my child would start running toward danger, I could have restrained her. After multiple visits at the beach, this might become my deontic, automatic, dynamic strategy (e.g., setting foot on the sand causes my arm to grab my child).

This means that the more “representationalist” picture of the continuous rational influence of planning, we claim, is subtly mistaken. For example, suppose I am playing table-tennis well. My context sensitive “precision settings” are all apt, no unexpected circumstances arise (alien invasions, etc.). In such circumstances, I harvest a flow of expected kinds of prediction errors. These get resolved, in broadly predictable ways, as play unfolds without pushing far up the processing hierarchy. But if “unexpected surprises” [for more on this distinction, see Yu and Dayan (2005)] occur, some errors are more fundamentally unresolved and get pushed higher. This provides the seed for re-organizing the precision of various likelihood mappings to lend more weight to different kinds of (internal, external, and action-involving) information. That, we suggest, is how we can remain constantly poised (e.g., Sutton’s (2007) compelling work on expert cricket) for nuance, even while behaving in the fast, fluent manner of a “habit machine.” In a deep sense, we exist in that moment as a habit machine – that is nonetheless constantly poised to become another transient machine should the need arise. This speaks to the coalition between representational and dynamic pathways illustrated in Figure 3.

Put another way, wherever possible, simple “habit” systems should guide behavior, dealing with expected prediction error fluently and fast. But where these fail, or where a change of context indicates the need, more and more knowledge-intensive resources (internal and external) can be assembled, via new waves of precision-weighting, to quash any outstanding prediction errors (i.e., free energy) – see Pezzulo et al. (2015) for a complete argument. Hence, we should not deny that there really is, in advanced minds, what representationalists correctly describes as “immense storage of causal knowledge” (Hohwy, 2019). But via moment by moment, self-organizing, free energy minimizing kinetics, we manifest as a succession of relatively special-purpose brain-body-world devices, strung together by those shifting but self-organizing webs of precision-weighting. Importantly, it is self-organizing around free energy that itself delivers the subsequent precision variations that recruit the “next machine.” There is no precision-master sitting atop this web, carefully deciding moment by moment just how to assign precision – there’s just the generative model itself.

### Second Worry

At this point a new version of representationalist worry may arise. For it may seem that precision estimates – the roots of each episode of re-structuring – are cognitively expensive and purely inner-model-bound. But this too – or so we have been arguing – is subtly mistaken. If we shift perspectives and timescales, we can see the human-built cognitive niche as itself a prime reservoir, both of achieved precision estimations and of tools for cheaply estimating precisions on-the-fly. And once learnt, they allow non-representation involving deontic action pathway (e.g., positioning cheap cues in the world such as warning triangles around a broken-down vehicle). These otherwise arbitrary structures attract attention and act as local proxies for precision [e.g., Roepstorff et al. (2010), Paton et al. (2013), Hutchins (2014)]. Urgent fonts, food packaging, and priestly robes all provide handy shortcuts for our precision estimating brains. Squint just a little bit and much of the human-built world – including all those patterned social practices such as stopping at red traffic lights – can be seen as a bag of tricks for managing precision estimation and epistemic trust (Fonagy and Allison, 2014; Parr and Friston, 2017a). And, as we behave in the present niche, we gradually alter it, “uploading” (Constant et al., 2018b) more and more of our individual and collective precision estimations into persisting (transmissible) material and social structures. These, in turn, alter the inner models that individuals need to command to negotiate their worlds.

### Third Worry

A final representationalist worry may be that fast and frugal, non-representational deontic action could simply not yield adaptive behavior in a highly volatile world like ours and thus may lead to suboptimal, maladaptive decision making (e.g., decision making that fails to generate action that succeeds with respect to environmental challenges); especially, if our generative models of precision are not apt for a volatile world (Parr et al., 2018a,b). Consequently, one should favor explanations based on rich reconstructing planning. This is a fair worry; a fair worry for humans in general, not for the dynamicists’ perspective, though. Indeed, humans learn to generate deontic actions that do not always lead to the “Machiavellian,” or perhaps “Darwinian” utility maximizing option relative to the current environment; we miss steps and fall down the stairs, forget to stop on the red, develop disorders such as PTSD that makes us misperceive threats, and generate many more maladaptive traits (Badcock et al., 2017; Cornwell et al., 2017; Peters et al., 2017). The tricks humans employ – to minimize the potential cost of normal maladaptive actions – is not to plan more “in the head,” but to plan more “in the world;” e.g., making sure that the synchronization of the traffic lights is consistent with the traffic flow at different hours of the day. This “planning the world” solution stabilizes the environment to enable the acquisition (i.e., learning through representationalist processes) of cheap deontic action shared among “cultural” conspecifics – “people enculturated like me, on a 9–5 schedule” (Constant et al., 2018a,b). Under that view, in certain situations, one can dispense with rich models that “stand-in for that world for the purposes of planning, reasoning, and the guidance of action” (Clark, 2015c, p. 6). In a word, for enculturated, deontically construed agents like us, the world is often “our shared” best model.

Now, it might be rightfully argued that the deontic route, even if it were to be non-representational, would still need representational processes to be acquired. We agree Borrowing from Shaun Gallagher (comment during the 2020 XPECT conference), it seems that what is at stake in the representation war is not whether there are or aren’t representations. Rather, the problem is to know whether they play a role in cognition or not. We are claiming that under active inference, it makes sense to assume that sometimes they do, and that sometimes (after sufficient learning) they don’t – at least, sometimes they don’t anymore. Given sufficient learning there might often be no need to infer what is the right (most likely) thing to do. One can then simply operate with the deontic route which involves committing to the sensory outcome afforded by the environment.

## Conclusion: Bury the Hatchet, or Use It to Carve a New Path(Way)

This paper offers a mathematically informed reading of generative models that could accommodate both richly representationalist and dynamicist views of cognition. We asked whether cognition under active inference is a richly representational or a dynamic process, reliant on simple cues and couplings. The answer was both. We first presented the representational model of action and perception that involves parameters A and B, whose evaluation, so we argued, corresponds to a process that would be deemed representational given a minimal definition of representations. Then, based on that definition, we described an alternative model for action that does not rest on A and B, and thus, could be viewed as bypassing representational processes. We then outlined – and responded to – some of the possible philosophical critiques of the deontic model.

What remains unclear, however, is whether particular cognitive processes underlying certain behavior are representational or not. To debate on that based on active inference, one ought to take the hatchet, and ask whether a new theoretical path(way) in generative models should be carved out. Indeed, any debate in the philosophy of cognitive science appealing to active inference (and its kin such as predictive processing, the Bayesian brain, and predictive coding) should clarify at the outset the manner in which the cognitive process of interest may be implemented in the generative model, and what are the components of the graphical model involved in the process. Clarifying at the outset the architecture of the generative model of interest should be sufficient to settle the technical dimension of the debate.

Such good practice would allow researchers to save time and energy by simply showing the manner in which the cognitive process of interest may be already implemented by existing neurocomputational architecture. In effect, the name of the game with active inference is to show how cognitive processes can be expressed as rearrangements or decompositions of the free energy functional and the architecture it implements in the graphical model; i.e., to show the manner in which the dynamics of the process of interest are built in the free energy formalism, that is, the manner in which the formalism unifies the process of interest as a special case of free energy minimization. Researchers could first explore the currently available generative models (relevant material is all freely available either in theoretical articles or as part of the Statistical Parametric Mapping 12 MATLAB toolbox). If the literature on the cognitive function of interest is not yet available, researchers could consider this a great opportunity for “getting their hands dirty” and proposing novel architectures that could account for the cognitive process and the associated behavior they want to characterize (Montague et al., 2012; Schwartenbeck and Friston, 2016). Ideally, these novel architectures should complement existing data on neuroanatomy and hierarchical neural dynamics (Friston et al., 2017b,c).

Finally, a limitation of the current paper is that we do not know yet what existing neurophysiology implements the dynamicist pathway we describe in Section 3. We have shown that dynamicism has a computational grip when implemented in the theory of active inference. However, one has yet to propose candidate neural correlates, which is a research enterprise for neuroscience made possible on the basis of an implementable processing theory such as the one discussed in this paper. Thus, despite the lack of empirical evidence, we consider settling the general active inference debate about representationalism a major development; since it is a first step toward scientifically informed debates on the representational nature of specific pathways, which could then feedback to further strengthen future philosophical discussions and inform research trajectories.

## Data Availability Statement

The original contributions presented in the study are included in the article/supplementary material, further inquiries can be directed to the corresponding author/s.

## Author Contributions

AC wrote the first draft. ACL modified the first draft. KF completed the final version. All authors contributed to the article and approved the submitted version.

## Conflict of Interest

The authors declare that the research was conducted in the absence of any commercial or financial relationships that could be construed as a potential conflict of interest.

## Footnotes

1 The word “entails” has a particular meaning here because, strictly speaking, the brain is viewed as performing inference under a generative model. Technically, the generative model is just a probability distribution over the (unobserved) causes of (observed) sensations. As such, the generative model only exists to the extent that neuronal dynamics maximize the evidence for that model (for a discussion see Ramstead et al., 2017).

2 Hidden states are sometimes called latent states and refer to variables that cannot be observed. There are effectively hidden behind observations and have to be inferred as random variables.

3 Approximate Bayesian inference should not be read as inference that is approximate; rather, it should be read as inference that can be realized. Indeed, as we will see later, exact Bayesian inference is a special case of approximate Bayesian inference when certain conditions are met.

4 This arbitrary probability distribution is variously called an approximate posterior, a recognition distribution or more simply a Bayesian belief.

5 Note that not every deontic action need be socially sanctioned; or rather, we humans have the ability to socially sanction ourselves. For instance, one might come up with a solo practice; a habit that is all mine, such as putting a sock on the doorknob to stop me leaving without my keys. After some time, I might forget why I put a sock on my doorknob, until the day where for some reason my habit fails me. Coming back home, realizing that I forgot my key, and explaining the situation to myself, I will realize that I forgot my key because I should have put the sock on the doorknob. I might think, “this is what someone ‘like me’ would and should have done!.” In the active inference literature, so far, sociality has been framed in two distinct ways (Vasil et al., 2020). (i) As the fact of having some Bayesian beliefs about the manner in which the social world causes certain inputs that we receive in certain social settings; beliefs that people external to me and “like me” would have (i.e., sharing similar generative models). (ii) As the learning of a deontic likelihood based on sensory entries that have been generated by people external to me and by people “like me.” In both cases, the two criteria for sociality are (i) the externality of the cause, and (ii) the fact that that cause is “like me.” There is a sense in which actions that are caused by me and that loop into the world so as to generate sensory entries suppose a cause that is external to me (e.g., my physical action), and that is caused by someone “like me” (i.e., me, literally). This allows, under active inference, to conceive of aspects of sociality (e.g., acting based on what “one should do”) that arise from interaction with oneself, even when alone. Such a “self-social inference” is rendered possible by the fact that my brain (my internal states) are conditionally independent from my active states (e.g., my body) despite being mine in the sense of generating reliable, recurrent, and predictable inputs (Constant et al., 2018b).

6 Example, when I see the sock on my night table, I put it in my pocket.

7 In the sense that there would be no opportunity to optimize the precision of the likelihood mapping between hidden states and outcomes that, in active inference, is usually associated with (covert) mental action and attention (Limanowski and Friston, 2018).

## Glossary of Terms and Expressions

**Table:**

**Expression**	**Description**
_{ s, o,π}_	External or latent states, sensory states or observations, and active states, plans or polices
_*P(o,s)*_	Generative model: i.e., a probabilistic specification of how external states cause sensory states
_*Q(s)*_	Posterior (Bayesian) belief about external states
_*Q(s_τ —π)*_	Predictive (Bayesian) belief about future states, under a particular policy or plan
_*F*_	Variational free energy – an upper bound on the surprisal of sensory states
_*G(π,τ)*_	Expected free energy – an upper bound on the surprisal of sensory states in the future
_ℑ(o)=–lnP(o)_	Surprisal or self-information
_*D[Q(s)——P(s)]=E_Q [lnQ(s)–lnP(s)]*_	Relative entropy or Kullback–Leibler divergence
_*P(o—π)=Ω*_	Deontic value, or likelihood of observations under a given policy
